# Tumor-Infiltrating T Cells in EBV-Associated Gastric Carcinomas Exhibit High Levels of Multiple Markers of Activation, Effector Gene Expression, and Exhaustion

**DOI:** 10.3390/v15010176

**Published:** 2023-01-07

**Authors:** Mikhail Salnikov, Martin A. Prusinkiewicz, Sherman Lin, Farhad Ghasemi, Matthew J. Cecchini, Joe S. Mymryk

**Affiliations:** 1 Department of Microbiology and Immunology, Western University, London, ON N6A 3K7, Canada; 2 Department of Pathology and Laboratory Medicine, Western University, London, ON N6A 3K7, Canada; 3 Department of General Surgery, Western University, London, ON N6A 3K7, Canada; 4 Department of Oncology, Western University, London, ON N6A 3K7, Canada; 5 London Regional Cancer Program, Lawson Health Research Institute, London, ON N6A 5W9, Canada; 6 Department of Otolaryngology, Western University, London, ON N6A 5W9, Canada

**Keywords:** Epstein–Barr virus, gastric cancer, TCGA, gene expression, immune landscape, lymphocyte infiltration, T cell function, neoantigens, tumor immunology, TCR repertoire

## Abstract

Epstein–Barr virus (EBV) is a gamma-herpesvirus associated with 10% of all gastric cancers (GCs) and 1.5% of all human cancers. EBV-associated GCs (EBVaGCs) are pathologically and clinically distinct entities from EBV-negative GCs (EBVnGCs), with EBVaGCs exhibiting differential molecular pathology, treatment response, and patient prognosis. However, the tumor immune landscape of EBVaGC has not been well explored. In this study, a systemic and comprehensive analysis of gene expression and immune landscape features was performed for both EBVaGC and EBVnGC. EBVaGCs exhibited many aspects of a T cell-inflamed phenotype, with greater T and NK cell infiltration, increased expression of immune checkpoint markers (BTLA, CD96, CTLA4, LAG3, PD1, TIGIT, and TIM3), and multiple T cell effector molecules in comparison with EBVnGCs. EBVaGCs also displayed a higher expression of anti-tumor immunity factors (PDL1, CD155, CEACAM1, galectin-9, and IDO1). Six EBV-encoded miRNAs (miR-BARTs 8-3p, 9-5p, 10-3p, 22, 5-5p, and 14-3p) were strongly negatively correlated with the expression of immune checkpoint receptors and multiple markers of anti-tumor immunity. These profound differences in the tumor immune landscape between EBVaGCs and EBVnGCs may help explain some of the observed differences in pathological and clinical outcomes, with an EBV-positive status possibly being a potential biomarker for the application of immunotherapy in GC.

## 1. Introduction

Epstein–Barr virus (EBV) is a gamma-herpesvirus that infects B lymphocytes and mucosal epithelial cells, influencing cellular differentiation and growth [1,2,3]. EBV uses a variety of immune evasion strategies to establish lifelong infections, via latency within B lymphocytes [4]. Indeed, it is estimated that 90% of all adults are infected with EBV [5]. Furthermore, EBV is associated with multiple types of cancers, including nasopharyngeal carcinomas, Burkitt’s and other lymphomas, and EBV-associated gastric adenocarcinomas (EBVaGCs) [6]. Overall, EBV infections account for 1.5% of all human cancers worldwide [7].

The etiology of EBV in gastric carcinomas (GCs) was first identified in 1990 by Burke et al. [8], but it would not be until two years later that Shibata and Weiss demonstrated not only the presence of the EBV genome within cancerous and dysplastic cells, but also its absence within surrounding healthy cells [9]. It is estimated that EBV is the causative agent of around 10% of all GC cases worldwide [10]. According to The Cancer Genome Atlas (TCGA), EBV-negative gastric cancers (EBVnGCs) consist of four subgroups: microsatellite-instable (MSI) tumors, genomically stable (GS) tumors, tumors with chromosomal instability (CIN), and tumors with DNA polymerase epsilon (POLE) mutations [11]. Importantly, EBVaGCs are molecularly and pathologically distinct entities from EBVnGCs, with higher survival rates, widespread promoter hypermethylation, increased CD4^+^ and CD8^+^ T cell infiltration, and higher levels of MHC-I and MHC-II expression [12,13,14,15,16,17].

The aim of this study was to compare the tumor immune landscape between EBVaGCs and EBVnGCs, with the goal of identifying differences with implications for disease diagnosis, prognosis, and treatment. Despite the differences in pathological and clinical outcomes, there have been few studies comparing the tumor immune landscapes of EBVaGCs and EBVnGCs [18,19]. Given the effectiveness of T cell targeting immune checkpoint inhibitors in cancer treatment [20], a detailed and systematic T cell-centric analysis was performed to compare EBVaGCs and EBVnGCs. RNA sequencing data and available pathological data from nearly 400 human GCs were employed to assess how the presence of EBV altered the immune landscape of GCs, including genes associated with T cell function, activation, and exhaustion. Significantly increased T cell infiltration, T cell receptor (TCR) repertoire diversity, effector gene expression, activation status, and exhaustion marker expression were observed in EBVaGCs when compared to EBVnGCs. Such observations indicated that the immune landscape of EBVaGCs was a T cell-inflamed phenotype [21,22]. Differences within the tumor immune landscape may be contributing factors in the decreased mortality associated with EBVaGCs and increased effectiveness of immunomodulatory treatments, such as immune checkpoint inhibitors.

## 2. Materials and Methods

### 2.1. Sample Collection and Ethics

All data from The Cancer Genome Atlas (TCGA) were downloaded via the Broad Genome Data Analysis Center’s Firehose server (https://gdac.broadinstitute.org/, accessed on 2 March 2017) or other publicly available sources as noted below; therefore, no ethical approval was needed.

### 2.2. Analysis of Cellular mRNA

Level 3 mRNA expression data for the TCGA GC dataset were sourced from the Broad Genome Data Analysis Center’s Firehose server (https://gdac.broadinstitute.org/, accessed on 2 March 2017). The GC RNA sequencing dataset comprised 30 EBV-positive, 353 EBV-negative (223 CIN, 73 MSI, 50 GS, and 7 POLE), and 35 normal control tissues [11]. The correlation of cellular gene mRNA expression and EBV status was performed with R’s built-in wilcox.test function with the conf.level parameter set to 0.95. The q-values were calculated for each comparison group using the qvalue function available from the similarly titled R library, with the false discovery rate (FDR) parameter set to 10%. All samples in this cohort were resected prior to treatment, avoiding any confounding effects of treatment.

### 2.3. Analysis of Immune Landscape Features

Selected immune landscape features based on precalculated multi-gene signatures were extracted from Thorsson et al. [23] for each of the TCGA GC samples and similarly analyzed as above via the sorting of the samples into EBVaGC, EBVnGC, and normal sample subsets. As not all individual TCGA samples had the data necessary for the calculation of each of these immune landscape features, these comparisons included only 30 EBV-positive and 353 EBV-negative GC samples.

### 2.4. Pathology-Based Analysis of TIL Infiltration and Spatial Organization

TIL infiltration and spatial organization features based on the precalculated digital pathological analysis of hematoxylin and eosin (H&E) stained diagnostic whole-slide images were extracted from Saltz et al. [24]. This was similarly analyzed as above via the sorting of the available sample data into EBVaGC and EBVnGC subsets.

For the EBVaGC pathological analysis, at least one representative slide was available for each case, with a minority of cases being frozen sections. The formalin fixed diagnostic slide was preferentially utilized. However, in 4 (of 30) cases, the poor quality of the diagnostic slide precluded analysis, and we chose not to use any of the frozen section tissue slides for consistency. Using QuPath [25], representative areas of tumor, immune, and stromal cells were annotated. The overall extent of the tumor was annotated, and all contours were reviewed by a pathologist (MJC). Within the defined tumor area, a cell detection algorithm in QuPath was utilized to enumerate all the cells and their standard nuclear and cytoplasmic features. The detected cellular features were used from the categorically defined manual contours for the basis of a cell classifier that stratified cells across a whole slide image in tumor, immune, and stromal cell populations. Given the heterogeneity of tumor and stain intensity between cases, we trained the object classifier on each case independently and reviewed all cases manually to ensure accuracy. Using the cell counts, the percentage of tumor cells was calculated for each case.

### 2.5. Correlation of EBV miR-BARTs with Cellular Gene Expression and Pathology Scores

The expression levels of the EBV microRNAs encoded by the Bam-HI A rightward transcripts (miR-BARTs) from the 30 EBV-positive TCGA GC samples were extracted from Ungerleider et al. [26]. Analyses of the gene expression correlations between the miR-BARTs in EBVaGC utilized TCGA level 3 mRNA expression data sourced from the Broad Genome Data Analysis Center’s Firehose server (https://gdac.broadinstitute.org/, accessed on 2 March 2017). The correlation of cellular gene mRNA expression with miR-BART expression or immune landscape gene signatures from Thorsson et al. [23] was performed with R’s built-in cor.test function with the conf.level parameter set to 0.95 and the method parameter set to spearman. For the pathology correlations, tumor, stromal, and TIL fractions were calculated by taking the cell counts of each group and dividing them by the total cell counts within the tumor mass. The correlation of miR-BART expression with stromal, TIL, and tumor fractions was performed with the cor.test function with the conf.level parameter set to 0.95 and the method parameter set to spearman, whereas 95% confidence intervals were calculated with the spearman.ci function available through the RVAideMemoire library.

### 2.6. Survival Analysis

Survival analyses utilized the TCGA overall survival (OS) data from Liu et al. [27]. The correlation of survival and cellular mRNA expression was performed via the sorting of the dataset into EBVaGC and EBVnGC subsets. Patients were dichotomized into high and low mRNA expression groups, with hazard ratios, significance, and 95% confidence intervals calculated via the coxph and Surv functions, both available via the survival library in R. A similar analysis was performed for EBVaGC samples based on viral miRNA expression.

## 3. Results

### 3.1. EBVaGCs Exhibit Greater Tumor Lymphocyte Infiltration as Compared to EBVnGCs

**Figure 1:**
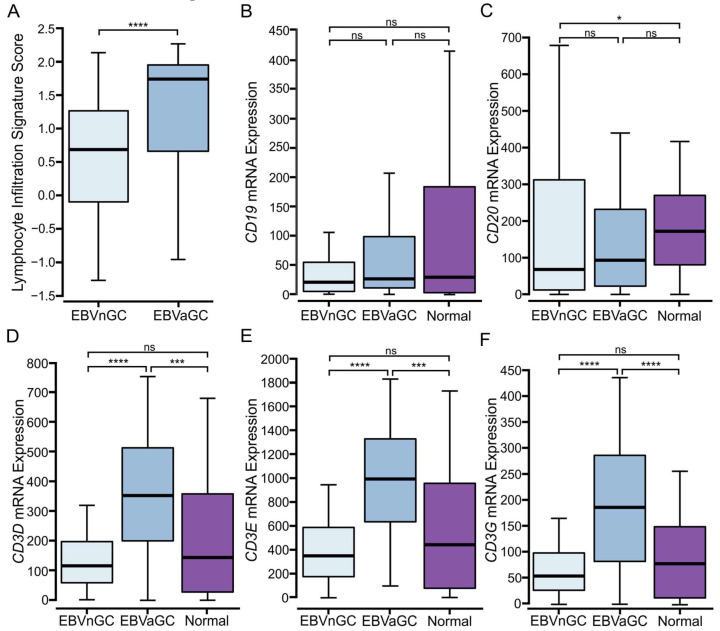
Analysis of tumor-infiltrating lymphocytes and subsets in EBV-positive and EBV-negative gastric cancers. (**A**) Comparison of lymphocyte infiltration signature score between EBV-positive (EBVaGC) and EBV-negative (EBVnGC) gastric cancers. (**B**–**F**) Expression of marker genes related to B cell (*CD19*, *CD20*) and T cell (*CD3D*, *CD3E*, *CD3G*) infiltration between EBV-positive, EBV-negative, and normal control samples. **** *p* ≤ 0.0001, *** *p* ≤ 0.001, * *p* ≤ 0.05, ns—not significant.

Recent developments in cancer immunotherapy have demonstrated the critical role of the immune system in suppressing malignancy. The phenotypes, numbers, and localizations of tumor-infiltrating lymphocytes (TILs) can provide insight into the tumor immune landscape and may be predictive of patient-specific responses to immunotherapeutic treatments [28]. To explore the tumor immune landscape in the context of GCs, we divided the five molecular subtypes of GCs into either EBVaGC or EBVnGC. TCGA Illumina HiSeq mRNA expression data for all 383 GCs were analyzed using a previously described lymphocyte infiltration signature score [23]. EBVaGCs exhibited significantly higher scores for lymphocyte infiltration as compared to EBVnGCs (Figure 1A). Absolute values suggest that EBVaGCs exhibit roughly three times higher TIL infiltration than their EBVnGC counterparts.

### 3.2. Higher Levels of T and NK, but Not B Lymphocytes Are Present in EBVaGCs

Relative proportions of B, T, and NK lymphocytes were determined via the relative expression of mRNAs encoding commonly accepted lineage-defining marker genes. Levels of *CD19* and *CD20* mRNA were used as measures of B cell infiltration. No differences in B cell infiltration were observed between EBVaGC and EBVnGC (Figure 1B,C); *CD3D, CD3E,* and *CD3G* were used as markers of T cell infiltration. Significantly higher levels of each of these three T cell markers were identified in EBVaGCs when compared to EBVnGCs (Figure 1D–F). Indeed, the absolute value of these genes was about three-fold higher in EBVaGCs. *KLRB1, KLRC1, KLRD1, CD160, XCL2, NCR1, KIR3DL1,* and *GNLY* were used for NK cell infiltration (Supplementary Figure S1). While the expression of these genes was generally lower than those encoding T cell markers, each of these marker genes was expressed at significantly higher levels in EBVaGC vs. in EBVnGC. Collectively, the results for T and NK cell markers are indicative of a more “immunologically hot” environment in EBVaGCs as compared to EBVnGCs [21].

### 3.3. Digital Pathology-Based Analysis Reveals Increased TIL Infiltration and Altered Spatial Organization of Lymphocytes in EBVaGCs Compared to EBVnGCs

**Figure 2:**
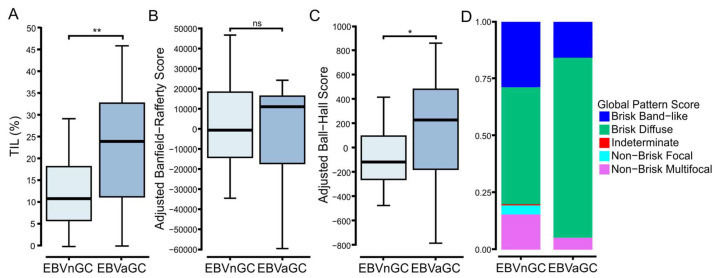
Pathology-based analysis revealed increased TIL infiltration and the altered spatial organization of lymphocytes in EBVaGCs compared to EBVnGCs. TIL levels and their distribution in hematoxylin and eosin stained diagnostic whole-slide images were spatially quantified by deep learning computational methods to determine the (**A**) overall TIL fraction, (**B**) Banfield–Rafferty score, and (**C**) Ball–Hall score. (**D**) Distributions of the pathologist assignments of the GCs into the indicated global TIL patterns. ** *p* ≤ 0.01, * *p* ≤ 0.05, ns—not significant.

The TCGA dataset also includes representative H&E stained diagnostic whole-slide images, which were used previously used for the spatial quantification and analysis of TILs by deep learning computational methods [24]. The extraction of this quantified pa-thology-based data for the GC samples revealed significantly higher levels of TILs in the TCGA EBVaGCs samples as compared to their EBVnGC counterparts (Figure 2A), which is in agreement with the increased TIL levels predicted based on gene expression markers (Figure 1A).

The pathology-based imaging data also assessed the clustering patterns of lymphocytic infiltration [24]. The spatial distribution of TILs within the complex tumor microenvironment may be critical for the immune-mediated resolution of cancer [29]. Notably, this adjusted analysis revealed a significant difference between the adjusted Ball–Hall score, which reflects the size of immune clusters, but not the Banfield–Rafferty score, which is related to the number of immune clusters (Figure 2B,C). This also translated into an increased fraction of EBVaGCs in the “Brisk Diffuse” category compared to EBVnGCs (Figure 2D). This category exhibits diffusely infiltrative TILs scattered throughout at least 30% of the area of the tumor [24].

### 3.4. Higher Levels of CD4^+^, CD8^+^ T Cells, and T Regulatory Cells Are Present in EBVaGCs

**Figure 3:**
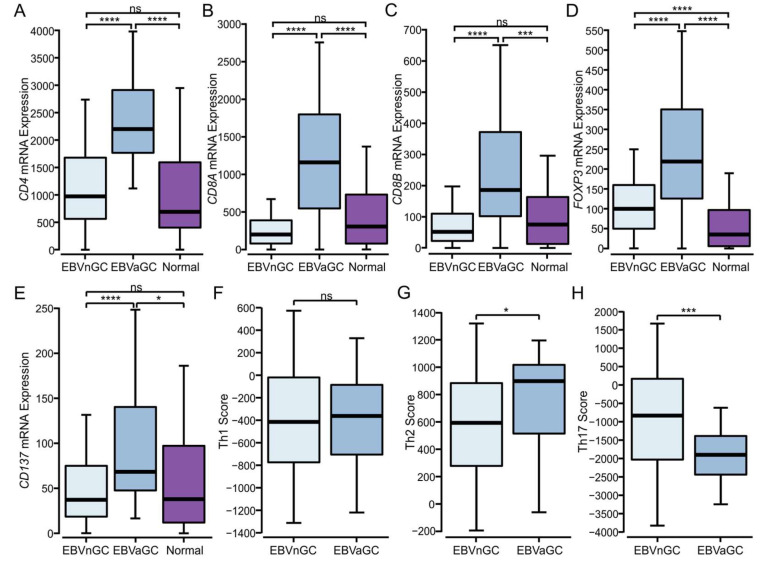
Analysis of T cell populations in EBV-positive and EBV-negative gastric cancer. (**A**–**E**) Expression of marker genes related to CD4+ helper (*CD4*), CD8+ cytotoxic (*CD8A* and *B*), Treg (*FOXP3*) cells, and *CD137* (TNFRSF9/4-1BB), an activation-induced costimulatory molecule present primarily on CD8+ T cells. (**F**–**H**) Comparison of CD4+ helper Th1, Th2, and Th17 signature scores between EBVaGC and EBVnGC samples. **** *p* ≤ 0.0001, *** *p* ≤ 0.001, * *p* ≤ 0.05, ns—not significant.

Given the high levels of T cells present in EBVaGCs, we assessed the relative proportions of CD4^+^ and CD8^+^ T cells and T regulatory cells (Tregs), based on the relative expression of the respective *CD4*, *CD8A* and *CD8B*, and *FOXP3* lineage-defining factors (Figure 3). EBVaGC samples exhibited the significantly increased expression of all four genes as compared to the EBVnGC samples, indicating increased infiltration by each of these T cell subtypes (Figure 3A–D). Additionally, *CD137* (4-1BB), an activation-induced costimulatory molecule primarily present on T cells, was expressed at significantly higher levels in the EBVaGCs as compared to EBVnGCs, suggesting a higher overall level of T cell activation in EBVaGC (Figure 3E). An analysis of CD4^+^ T helper signatures from Thorsson et al. [23] suggests that GCs are biased towards a humoral Th2-mediated response, rather than a cell-mediated Th1 response or a general inflammatory Th17 response. More specifically, the immune response in EBVaGCs was shifted more towards a Th2 response and away from a Th17 one as compared to the EBVnGCs (Figure 3), which aligns with the competitive nature of the two responses [30].

### 3.5. EBVaGCs Express Higher Levels of T Cell Effector Molecules as Compared to EBVnGCs with Characteristics of a T Cell-Inflamed Phenotype

**Figure 4:**
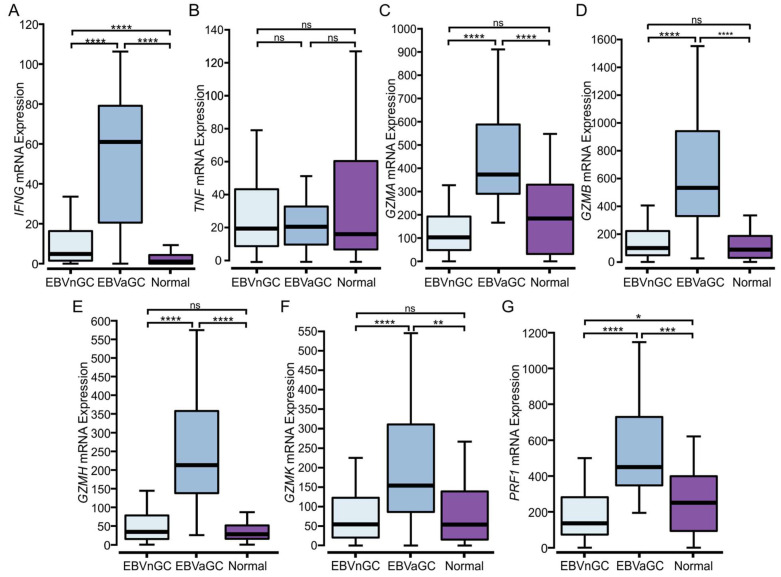
Transcript levels of lymphocyte effector molecules in EBV-positive and EBV-negative gastric cancers. (**A**–**G**) Expression of marker genes related to activated cytotoxic CD8+ T cells, including IFN-γ (*IFNG*), *TNF*, granzyme A (*GZMA*), granzyme B (*GZMB*), granzyme H (*GZMH*), granzyme K (*GZMK*), and perforin (*PRF1*). **** *p* ≤ 0.0001, *** *p* ≤ 0.001, ** *p* ≤ 0.01, * *p* ≤ 0.05, ns—not significant.

Immune hot tumors are associated with the presence of activated cytotoxic CD8+ T cells, which produce a variety of proinflammatory cytokines, including IFN-γ and TNF [21,22]. Whereas IFN-γ mRNA was expressed at significantly higher levels in EBVaGC compared to EBVnGC, no significant difference was observed for TNF mRNA expression (Figure 4A,B). EBVaGCs also expressed significantly higher levels of transcripts encoding cytotoxic mediators, including granzyme A (*GZMA*), granzyme B (*GZMB*), granzyme H (*GZMH*), granzyme K (*GZMK*), and perforin (*PRF1*) as compared to EBVnGCs (Figure 4 C–G). These observations indicate that CD8^+^ T cells exhibited greater activation and effector molecule production in EBVaGCs, in addition to their increased levels of tumor infiltration (Figure 3B,C).

**Figure 5:**
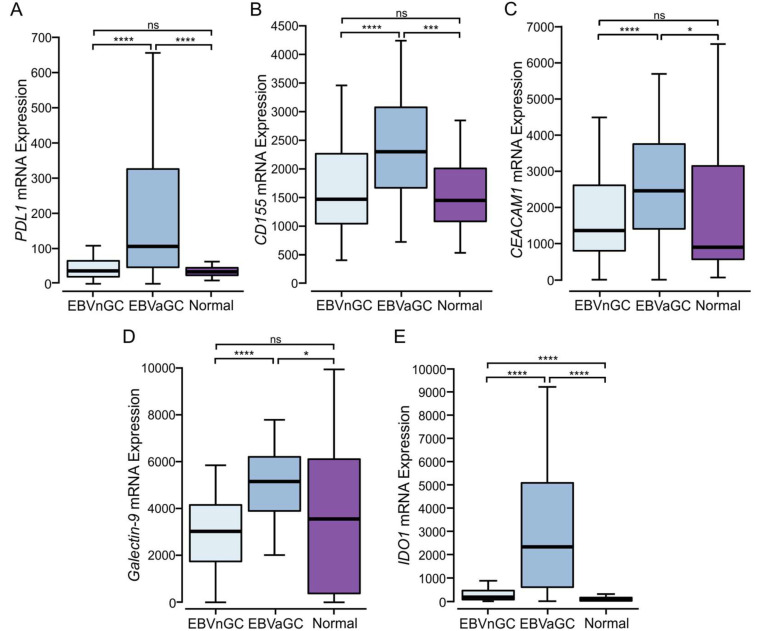
Transcript levels of tumor immune evasion genes in EBV-positive and EBV-negative gastric cancers. Normalized RNA-Seq data for genes associated with tumor immune evasion, including (**A**) *PDL1* (CD274), (**B**) *CD155*, (**C**) *CEACAM1*, (**D**) *LGALS9* (galectin-9), and (**E**) *IDO1*. **** *p* ≤ 0.0001, *** *p* ≤ 0.001* *p* ≤ 0.05, ns—not significant.

The increased T cell infiltration and higher gene expression levels of effector molecules in EBVaGCs may be indicative of a T cell-inflamed tumor phenotype [21]. This is further supported by the increased expression of the anti-tumor immunity genes *PDL1*, *CD155* (PVR), *CEACAM1*, *LGALS9*, and *IDO1* (Figure 5), all of which antagonize T cell responses via their interaction with inhibitory receptors. PDL1 and IDO1 are also well established markers of T cell-inflamed tumors [21,22].

### 3.6. EBVaGCs Express Higher Levels of Immune Checkpoint Markers

**Figure 6:**
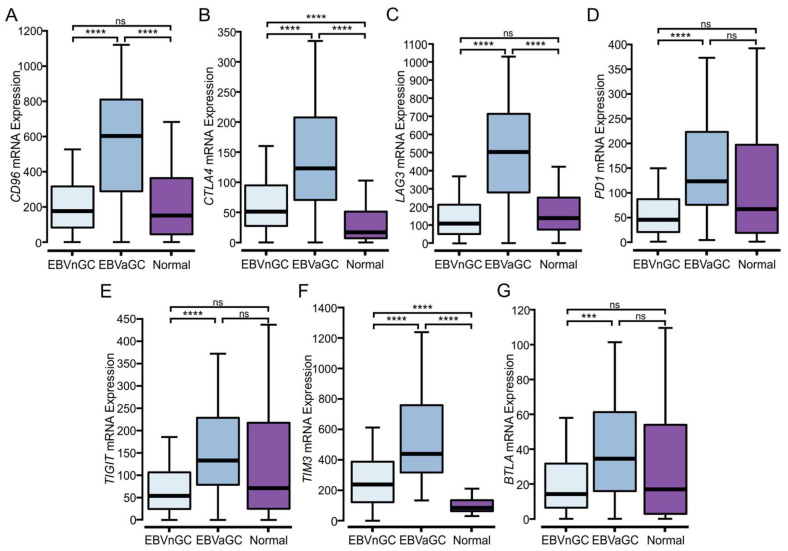
Analysis of immune checkpoint markers in EBV-positive and EBV-negative gastric cancers. (**A**–**G**) Expression of genes related to T cell exhaustion markers *CD96*, *CTLA4*, *LAG3*, *PD1* (PDCD1), *TIGIT*, *TIM3* (HAVCR2), and *BTLA* in EBV-positive (EBVaGC), EBV-negative (EBVnGC), and normal control samples. **** *p* ≤ 0.0001, *** *p* ≤ 0.001, ns—not significant.

Once activated, T cells upregulate the expression of multiple cell surface receptors that negatively regulate their proliferation and activation [31,32]. Such “checkpoint” marker genes include *CD96*, *CTLA4*, *LAG3*, *PD1*, *TIGIT*, *TIM3*, and *BTLA*, all of which are generally expressed at higher levels in T cell-inflamed tumors [23]. Notably, these checkpoint genes are significantly upregulated in EBVaGCs as compared to EBVnGCs (Figure 6A–F). This indicates a greater T cell exhaustion signature within EBVaGCs, which is associated with the sustained CD8+ T cell activation phenotype characteristic of immunologically hot tumors.

### 3.7. Comparison of the T Cell Receptor Repertoire between EBVaGC and EBVnGC

**Figure 7:**
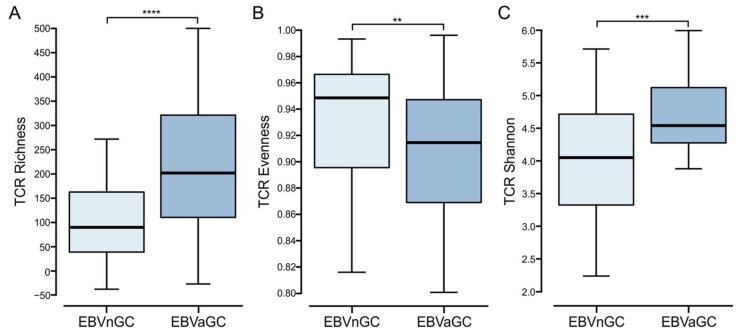
Comparison of the T cell receptor (TCR) repertoire between EBV-positive and EBV-negative gastric cancers. (**A**) Comparison of unique TCR sequences in the TCR repertoire (richness). (**B**) Comparison of the distribution spectrum of TCR sequences, reflecting the relative abundance of individual T cell clones (evenness). (**C**) Comparison of clonal diversity weighted by the abundance of each complementarity-determining region 3 (Shannon entropy). **** *p* ≤ 0.0001, *** *p* ≤ 0.001, ** *p* ≤ 0.01, ns—not significant.

There is increasing evidence that the analysis of the TCR repertoire may serve as a biomarker of immune response quality in cancer patients [33]. Stemming from this, the TCR repertoire was compared between EBVaGCs and EBVnGCs using signatures from Thorsson et al. [23] (Figure 7). The TCR richness score was significantly higher in EBVaGC than in EBVnGC, indicating an increased number of unique TCR sequences. TCR evenness, which represents the relative abundance of individual T cell clones, was lower in EBVaGC as compared to EBVnGC. The Shannon entropy, which represents the clonal diversity weighted by the abundance of each complementarity-determining region 3 (CDR3), was also significantly higher in EBVaGC as compared to EBVnGC, indicating a greater diversity of unique T cell receptors in EBVaGC. Taken together, the TCR repertoire in EBVaGC is wider and more diverse as compared to EBVnGC.

### 3.8. EBV-Encoded miRNAs Strongly Impact the Tumor Immune Microenvironment

A total of 99% of all virally derived polyadenylated transcripts in EBVaGCs are from the 44 miR-BARTs [34]. The viral miR-BARTs are also highly expressed, representing >10% of the total pool of miRNAs in EBVaGCs [35]. These pleotropic regulators are known to target critical viral and cellular genes, modulating nearly every aspect of the cancer phenotype [36].

**Figure 8:**
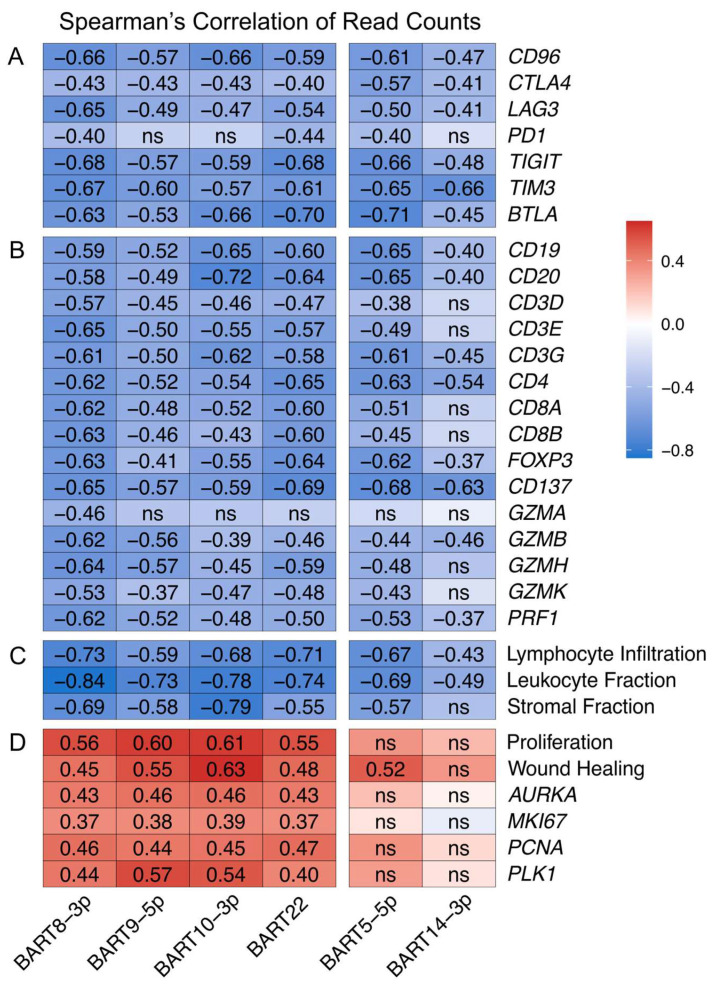
A subset of EBV-encoded miRNAs are strongly correlated with decreased tumor immune involvement. Spearman’s correlation matrix of the expression of the indicated EBV miR-BARTs and (**A**) T cell exhaustion marker genes, (**B**) B and T cell gene markers and effector gene markers, (**C**) gene signatures predicting tumor infiltration by lymphocytes or leukocytes and stromal content, and (**D**) gene signature for cell proliferation or proliferation marker genes. Unless otherwise indicated, all changes were statistically significant with *p* ≤ 0.05. ns—not significant.

Given the importance of the BARTs, we performed a Spearman’s correlation analysis to determine if any of the miR-BARTs were associated with the high expression of T cell exhaustion marker genes identified in Figure 6. The miR-BARTs 5-5p, 8-3p, 9-5p, 10-3p, 14-3p, and 22 were strongly and significantly correlated with the decreased expression of nearly all of the T cell exhaustion markers (Figure 8A). This strong negative correlation extended to virtually all T and B cell markers (Figure 8B) and extended to gene signatures predicting tumor infiltration by lymphocytes or leukocytes and stromal content (Figure 8C). Notably, of all the EBV-encoded miRNAs, only these six BARTs significantly and consistently exhibited a negative correlation with immune cell genes and landscape features (Table S1). In addition, none of the expression levels of the other EBV-encoded mRNAs was significantly and extensively correlated with immune cell gene or landscape features (Table S2). These decreases indicate that EBVaGCs expressing high levels of these specific miR-BARTs likely exhibit a tumor immune microenvironment closer to an immune excluded or immune desert phenotype [37,38].

Correlation with other immune signatures from Thorsson et al. [23] revealed a strong and significant positive correlation for miR-BARTs 8-3p, 9-5p, 10-3p, and 22, but not 5-5p or 14-3p, with proliferation and wound healing signatures, as well as individual well characterized gene markers for proliferation such as *AURKA*, *MKI67*, *PCNA*, and *PLK1* [39] (Figure 8D). As the wound healing signature is based on the fibroblast response to serum, this also reflects increased cell proliferation [40]. These correlations are unlikely to simply be related to tumor cell fractions, as many other BARTs are expressed at similar or even higher levels (Table S3) yet are not consistently and significantly correlated with these proliferation markers (Table S1). Collectively, these data support that the high expression of miR-BARTs 8-3p, 9-5p, 10-3p, or 22 is associated with highly proliferative tumors.

The corresponding TCGA H&E pathology slides were also examined for any correlations between tumor composition in terms of the tumor cell fraction, TIL fraction, and stromal fraction with miR-BART expression. There was also a positive correlation with tumor purity and a negative correlation with the TILs for all six of these EBV-miRNAs (Table S4). However, virtually none of these correlations was significant, potentially due to biases in the sampling between the tissues collected for pathological diagnosis and molecular analysis as described by Saltz et al. [24].

### 3.9. Clinical Impact of the Tumor Immune Microenvironment and miR-BART Expression

We also assessed the impact of the expression of the various immune-related genes on patient overall survival (Table S5). We dichotomized the EBVaGC and EBVnGC datasets into high and low expression subsets and calculated their relationship with overall survival. Very few significant correlations were observed. However, the high expression of many immune genes in EBVnGCs exhibited trends towards increased survival. In contrast, the converse was seen in EBVaGCs. These results suggest that the immune microenvironment is indeed different between EBVaGCs and EBVnGCs. A similar analysis was performed based on the miR-BART expression in EBVaGC (Table S5). While no significant correlation was identified, the higher expression of miR-BARTs generally trended towards increased survival. 

## 4. Discussion

Despite the well understood differences in the pathological [41,42] and clinical outcomes [43,44,45] between EBVaGCs and EBVnGCs, comparisons of their respective tumor immune microenvironments are generally restricted to a subset of markers in a subset of subtypes [46,47,48,49,50]. There is ever increasing evidence that patient outcomes are greatly impacted by the interactions of tumors with their local immune microenvironments [51]. Given the presence of foreign viral antigens in EBVaGC, but not EBVnGC, the high level of MHC-I and MHC-II expression [15,16] and the tremendous impact of T cell-specific immune checkpoint inhibitors on cancer treatment [20], we performed an in-depth, T cell-centric analysis to compare the tumor immune landscapes of EBVaGCs and EBVnGCs. Our analysis employed data from a treatment-naïve GC cohort obtained from TCGA. Since the data pertain to RNA expression levels, they may not fully reflect protein expression, as they do not take into account the influence of translational and posttranslational regulation [52]. To address this limitation, we have compared our systematic transcriptomic analysis with the available pathological data from this cohort [24] and actual protein expression data from previous studies more narrowly focused on individual genes.

For the initial survey of the tumor immune landscape, established immune lineage-specific markers and signatures were used [23,53,54,55]. We observed that EBVaGCs exhibited significantly higher lymphocyte infiltration than EBVnGCs, including both B and T cells (Figure 1). CD4+ helper T cells, CD8+ cytotoxic T cells, Tregs, and NK cells were much more abundant within the EBVaGC tumors (Figure 3 and Supplementary Figure S1). This agrees well with the existing literature [50,56,57,58,59] and TIL estimates from the pathological assessment of this cohort (Figure 2). Differences in the spatial distributions of the TILs were also noted between EBVaGCs and EBVnGCs, reflecting an increased pattern of diffusely infiltrative TILs throughout a large fraction of the tumor area (Figure 2). We also found that Th2 cells were the dominant CD4+ helper T cell subtype within both EBVaGCs and EBVnGCs (Figure 3). This also agrees with the existing literature [60,61]. Interestingly, within EBVaGCs, the response is further biased towards Th2 and away from Th17. The bias towards the Th2 subtype within EBVaGCs could lead to alternative pathway activation in tumor elimination [62] and has been associated with a better prognosis [63].

The higher T cell infiltration observed in EBVaGC tumors was accompanied by higher CD137 (4-1BB) gene transcript levels, indicative of increased T cell activation. There were higher levels of T cell effector protein and cytokine production (Figure 4). Indeed, EBVaGCs express significantly higher levels of mRNAs encoding IFN-γ, perforin, and granzymes A, B, H, and K as compared to EBVnGCs, which is in agreement with the existing pathology-based studies [56,64]. Notably, TNF did not show significant differences in expression between EBVaGCs, EBVnGCs, or normal control samples.

Based on our observation of the higher immune infiltration and expression of T cell effector molecules, it is evident that EBVaGCs share many of the same characteristics with tumors exhibiting a T cell-inflamed phenotype [21,22]. Our analysis also shows that EBVaGCs also exhibit higher levels of both *PDL1* and *IDO1* (Figure 5). The expression of these anti-tumor immunity molecules is also characteristic of a T cell-inflamed phenotype [65]. Our bioinformatic observations are supported by many other reports that EBVaGCs express higher levels of PDL1 protein [66,67,68,69,70,71]. In vitro work has also definitively shown that many EBVaGC cell lines expressed high levels of PDL1, which functionally contributed to the suppression of T cell proliferation [71].

In addition to *PDL1*, EBVaGCs express higher levels of *CD155/PVR*, *CEACAM1*, and *galectin-9* mRNAs (Figure 5), which represent known anti-tumor immunity genes that antagonize T cell responses. This is accompanied by the increased expression of multiple T cell immune checkpoint molecules within EBVaGCs, another characteristic of a T cell-inflamed phenotype [21,22]. The expression of *CD96, CTLA4, LAG3, PD1, TIGIT, TIM3*, and *BTLA* is all significantly higher in EBVaGC when compared to EBVnGCs (Figure 6). Previous work has shown that high PD1, LAG3, and TIM3 expression, detected by immunohistochemistry, was also strongly associated with EBV positivity [72]. The data obtained in our analysis falls in line with previous transcriptomic studies where it was observed that *CD96, CTLA4, LAG3, PD1, TIGIT*, and *TIM3* are upregulated in EBVaGCs when compared to EBVnGCs in several different cohorts [18,73].

The coordinated and high expression of such a wide array of T cell checkpoint molecules suggests that EBVaGCs could be particularly responsive to immune checkpoint inhibitors compared to all other types of GCs. Currently, PD-1 inhibitors such as nivolumab and pembrolizumab are approved for the treatment of GCs [74,75]. However, these do not take EBV status into consideration. Many other immunotherapeutics targeting immune checkpoint inhibitory receptors are undergoing clinical trials to determine their effectiveness in a variety of cancer types [76]. An expanded range of immune checkpoint inhibitors could be particularly useful in EBVaGC treatment considering the observation that high CD96 levels have been associated with improved patient survival [73]. Furthermore, combination therapies have shown promising results for improving overall survival for GC patients [77], and with more potential targets, novel combinations may prove to be even more advantageous. For example, the recent approval of relatlimab for the treatment of metastatic melanoma as a first-in-class drug targeting LAG3 [78] opens the door for trials with EBVaGC, which are readily justified by the high expression of LAG3 in EBVaGC.

Unexpectedly, we identified a strong negative association between six of the EBV miR-BARTs and virtually all aspects of the tumor immune microenvironment (Figure 8A–C). In contrast to a previous study, none of the miR-BARTs was positively correlated with *PDL1* expression [79]. Impressively, the high expression of these virally encoded miRNAs not only led to a loss of immune infiltration, but a concomitant reduction in stromal content, indicative of an increase in tumor purity. The expression of miR-BARTs 8-3p, 9-5p, 10-3p, and 22 was strongly correlated with an increase in tumor proliferation markers and signatures (Figure 8D). This is supported by experimental studies showing that each of these miRNAs induced proliferation in cell line models [80,81,82,83,84]. Importantly, this high proliferation index is somehow antagonistic to immune infiltration. Indeed, enhanced tumor cell proliferation appears to drive an increase in tumor purity, reducing immune infiltration, leading to tumors with phenotypes that are effectively immune excluded or immune desert situations. This subset of EBVaGCs may consequently be less responsive to immune checkpoint inhibitors. In contrast, neither miR-BART 5-5p nor 14-3p is strongly correlated with tumor proliferation, and they presumably function via an alternative mechanism to reduce tumor immune infiltration. These results suggest that the high levels of expression of these specific miR-BARTs may negatively impact tumor responses to immune checkpoint inhibitor therapy, given the reduced levels in TILs. Intriguingly, EBVaGCs expressing higher levels of miR-BARTs trended towards increased survival (Table S5). This might reflect the antigenic activity of highly expressed viral products in post-treatment immune responses or an increased sensitivity to therapy.

Differences between EBVaGCs and EBVnGCs were also identified in NK cell infiltration (Supplementary Figure S1). These results are supported by multiple studies using immunohistochemistry [59,64,85]. With the emergence of NK cell-based anti-cancer therapies, novel strategies have been developed in combatting tumors [86]. Much like T cells, NK cells can be engineered to express chimeric antigen receptors (CARs) specific against antigens expressed on the surface of tumor cells [87]. CAR NK cells have been previously employed in gastric cancers with some level success [88,89]. Other possibilities include therapies targeting NK cell inhibitory receptors or pathways that reactivate NK cell responses. For example, monalizumab, an anti-KLRC1 blocking monoclonal antibody, has been tested in several clinical trials, either as a single agent or in combination with other therapeutics [90,91]. The significantly higher expression of KLRC1 in EBVaGC as compared to EBVnGC indicates that this therapy may be a beneficial treatment in EBVaGCs.

Taken together, this study provides clear evidence of the distinct tumor immune landscapes of EBVaGC and other GCs, with EBVaGC exhibiting many of the hallmark characteristics of a T cell-inflamed phenotype. Notably, we have identified multiple mechanisms negatively regulating the anti-tumor immune response that are significantly upregulated in a majority of EBVaGC cases. These exhaustion markers may serve as useful biomarkers for prognosis, as well as novel targets for immunotherapies.

## Data Availability

Not applicable.

## Supplementary Materials

The following supporting information can be downloaded at: https://www.mdpi.com/article/10.3390/v15010176/s1, Figure S1: Transcript levels of NK cell marker genes in EBV-positive (EBVaGC) and EBV-negative (EBVnGC) gastric cancers, Table S1: Spearman’s Correlation Analysis of EBV miRNA Expression and Selected Immune Features/Genes, Table S2: Spearman’s Correlation Analysis of EBV mRNA Expression and Selected Immune Features/Genes, Table S3: Ranked Average Expression of EBV-Encoded miRNAs, Table S4: Correlation of Expression of Selected EBV miRNAs with Tumor Fractions Determined by Pathological Assessment, Table S5: Hazard Ratio of Expression of Selected Immune Related Genes and miR-BART Expression Levels with Patient Overall Survival.
