# Supplementary material for: Tumor-Infiltrating T Cells in EBV-Associated Gastric Carcinomas Exhibit High Levels of Multiple Markers of Activation, Effector Gene Expression, and Exhaustion
Source: Viruses. 2023 Jan 7;15(1):176. doi: 10.3390/v15010176 (PMC9860965; doi:10.3390/v15010176)
Supplement: Supplementary file 1 [file viruses-15-00176-s001.zip › viruses-2098222-Figure S1-for proof.pdf]

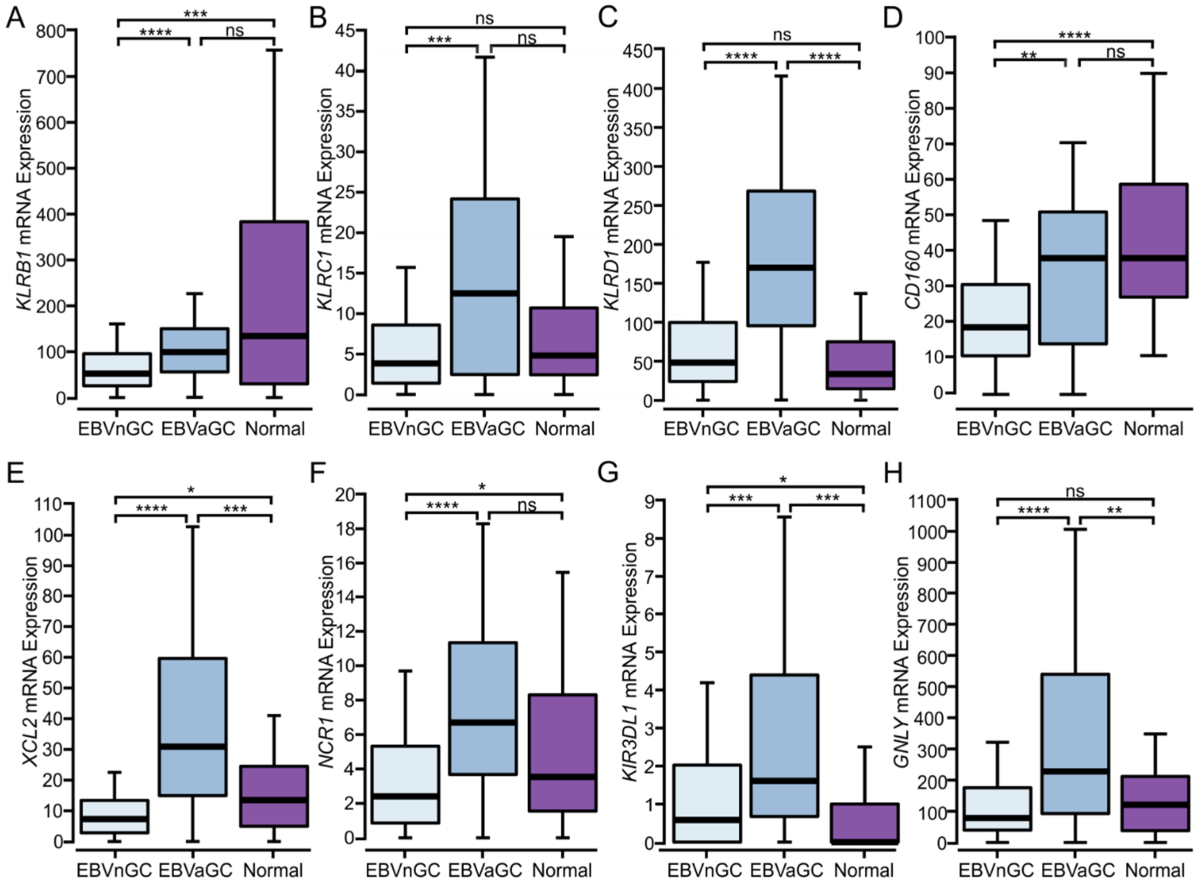

Figure S1. Transcript levels of NK cell marker genes in EBV-positive (EBVaGC) and EBV-negative (EBVnGC) gastric cancers. **** $p \leq 0.0001$, *** $p \leq 0.001$, ** $p \leq 0.01$, * $p = 0.05$, ns - not significant.
